# On the use of sham transcutaneous spinal cord stimulation in spinal cord injury clinical trials

**DOI:** 10.1093/brain/awaf040

**Published:** 2025-02-04

**Authors:** John L K Kramer, Tania Lam, Fabio M V Rossi, Judy Illes

**Affiliations:** Department of Anesthesiology, Pharmacology, and Therapeutics, Faculty of Medicine, University of British Columbia, Vancouver, Canada, BC V5Z 1M9; International Collaboration on Repair Discoveries (ICORD), Faculty of Medicine, University of British Columbia, Vancouver, Canada, BC V5Z 1M9; Vancouver Coastal Health Research Institute, Vancouver Coastal Health, Vancouver, Canada, BC V5Z 1M9; International Collaboration on Repair Discoveries (ICORD), Faculty of Medicine, University of British Columbia, Vancouver, Canada, BC V5Z 1M9; Vancouver Coastal Health Research Institute, Vancouver Coastal Health, Vancouver, Canada, BC V5Z 1M9; School of Kinesiology, Faculty of Education, University of British Columbia, Vancouver, Canada, BC V6T 1Z1; School of Biomedical Engineering and Department of Medical Genetics, University of British Columbia, Vancouver, Canada, BC V6T 1Z3; Neuroethics Canada, Division of Neurology, Department of Medicine, University of British Columbia, Vancouver, Canada, BC V6T 2B5

## Abstract

Kramer *et al.* examine the reasons put forth by investigators for excluding a sham condition in trials of neuromodulation therapies for individuals with spinal cord injury. They conclude that current dogma does not justify this design, and emphasize the need for future trials to include appropriate controls.

The excitement surrounding neuromodulation to improve health outcomes in individuals with spinal cord injury is palpable. Go to a conference or pick up an issue of a preferred scientific journal, and you will come to learn about progress and emerging applications in the field. The sheer volume of publications relevant to spinal cord injury and spinal cord stimulation—one form of neuromodulation—paints a clear picture of enthusiasm, increasing by more than 18-fold since 2012. Indeed, by now, the entire field has likely seen, either first-hand or by way of video, the profound and sensational effects of neuromodulation, evidenced as voluntary movement in individuals who are otherwise completely or nearly completely paralysed.

For a condition lacking an intervention that restores neurological function, the dawn of neuromodulation brings a renewed sense of optimism, giving hope to both individuals and their care providers directly affected by spinal cord injury, as well as researchers who have long searched for answers. Accompanying this optimism is a growing concern: how will the field of spinal cord injury shift from demonstrating the acute, promising effects of neuromodulation to meaningful and lasting functional benefits? A necessary step in this process is well designed clinical trials—a concept with which the field of neuromodulation, applied in other conditions (e.g. chronic pain), has long struggled.

The answer to this question appears in a publication in *Nature Medicine*^1^ last year (2024) showcasing benefits of transcutaneous spinal cord stimulation on upper extremity function in individuals with tetraplegia in an open label, unblinded trial design. The authors of the UP-LIFT trial proposed several lines of rationale to justify the limitations of excluding a control sham condition, which we critically appraise here.

##  

### Sham transcutaneous spinal cord stimulation is not impossible

Authors of the neuromodulation trial in focus proposed that the ‘feasibility of blinding becomes challenging, if not impossible, when the treatment requires the participants to perceive the electrical fields produced by the neuromodulation therapies’.^1^ While challenges of blinding transcutaneous spinal cord stimulation are unquestionable, other forms of neuromodulation that are accompanied by sensory feedback are routinely administered in a blinded fashion. For example, there exists sham transcranial magnetic stimulation, which is accompanied by discrete sensory experiences readily perceived by participants.^2,3^

The notion that ‘sham transcutaneous spinal cord stimulation is impossible’ also ignores published spinal cord injury trials that have used inert forms of stimulation as sham.^4^ These are, in theory, ideal shams with regard to maintaining participant as well as examiner blinding. The most accurate depiction of current knowledge is that sham transcutaneous spinal cord stimulation is untested: to our knowledge, no study has ever attempted to develop and validate a sham approach applicable in individuals with spinal cord injury.

### Sham transcutaneous spinal cord stimulation is not ethically questionable

Placebo or sham conditions are ethically permitted in research under three general conditions,^5^ which are summarized in Fig. 1. Most obviously, the use of placebo or sham depends on whether there is an existing treatment that, if withheld, would be unethical. Next is the risk that a participant faces by being randomized to placebo or sham. Risk is inherent to participation in research (i.e. the effect of active intervention is not yet known and it may cause harm). Putting participants at risk is ethically acceptable under the basic premise that the benefits of understanding the efficacy of an intervention outweigh harms, and that in order to understand the efficacy, the control group must undergo the same study procedures as the treatment group. The willingness of participants to take on this risk to understand the effects of a novel intervention reflects the altruism of their engagement in clinical trials.

**Figure 1:**
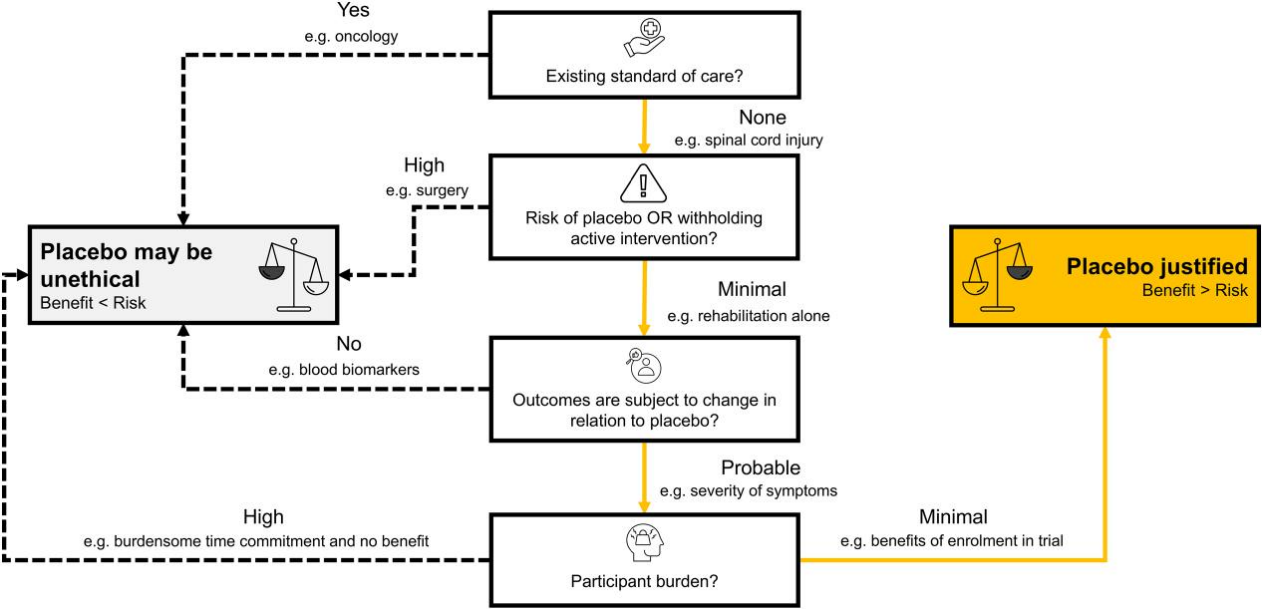
**Decision pathway for the inclusion of a sham condition in spinal cord injury clinical trials.** We argue that a trial testing transcutaneous spinal cord stimulation follows the orange path and meets the criteria for ethical justification of a placebo/sham condition. The dotted lines and examples may justify the exclusion of a placebo condition depending on the active intervention. Icons are from the Noun Project and were used through a Creative Common Attribution License, CC-BY 3.0 (Created by Hermawan: ‘risk’, fatimahazzahra: ‘standard of care’, WR Graphic Garage: ‘burden’, Icon Sea: ‘scale’, and Naya Putri: ‘subject to change’).

Tolerance for risk is, however, much lower for individuals in the placebo or sham condition as they typically stand not to benefit from the intervention. The challenge this creates is often exemplified in trials that involve a surgical condition, where sham surgery is deemed too great of a risk to warrant inclusion. A relevant example in the field of spinal cord injury relates to intrathecal delivery of saline—a trial procedure that mimics the delivery of active treatment. While putting participants at risks associated with lumbar puncture, such a procedure permits an unbiased comparison of placebo and active treatment (i.e. both groups went through identical study procedures).^6^

Along similar lines, a placebo or sham condition may not be included because of concerns related to withholding an active intervention that is deemed too great of a risk due to the serious nature of adverse effects (e.g. treatment-resistant depression and risk of death by suicide). Furthermore, trials need to consider if outcomes used to track improvements in a given function warrant a placebo or sham—i.e. whether they are expected to change unrelated to intervention. This is often the rationale for excluding a placebo condition in oncology trials, where there is little expectation that the tumour will respond to an inert intervention.^7^

In the context of transcutaneous spinal cord stimulation in chronic spinal cord injury, there is little reason to expect that sham intervention poses an ethical problem. There is no effective treatment for spinal cord injury, and various forms of electrical stimulation have been applied to individuals with spinal cord injury for many decades as part of rehabilitation. Therefore, whatever sham parameters are ultimately adopted, few deleterious side effects are reasonably anticipated. Moreover, after the first year of spinal cord injury, there is also no reason to expect that withholding active stimulation puts a participant at risk of not achieving a benefit if the intervention is applied at a later time. We do note, however, that an ethical case for excluding a sham condition could be made for a trial using transcutaneous spinal cord stimulation in the acute phase of injury, if there were rational grounds on which to propose that stimulation leveraged ongoing neuroplasticity thought to occur during this period.

Outcomes important to individuals with spinal cord injury (e.g. functional autonomy, walking endurance) are also unquestionably impacted by placebo. This is clearly evidenced in published trials that have included a sham condition (e.g. normoxic air and significant improvements in 6-min walk distances).^8^ Placebo effects comprise a number of factors, including biases of examiners and positive expectations of the participant. The effect of co-interventions is arguably the most important to consider in the context of neuromodulation trials involving individuals with spinal cord injury. This relates to the common practice of pairing stimulation with task-specific rehabilitation, which itself drives improvements in function. Less obvious but no less impactful on the outcomes of a clinical trial is that, by virtue of enrolling in a trial aimed at improving a specific function, participants are likely to engage more in activities associated with that function in their daily lives. This gives rise to benefits over time, known as the Hawthorne effect. These, and other factors such as regression towards the mean, can only be resolved from active treatment effects through the inclusion of a sham condition.

### Balancing practical and trial design issues

At the intersection of what is ethical and what should be done scientifically lies practical constraints. Spinal cord injury trials of any kind are unquestionably challenging. This is true from the perspective of the researcher (e.g. small population to recruit from, diverse injury characteristics, oversight and difficult study procedures) and, even more so, the participant (e.g. time commitment to engage in research for potentially small gains in function). Adding a sham condition makes trials many times more challenging to complete. This raises an ethical dilemma not covered by the aforementioned tenets of including placebo/sham: does the burden of participating in clinical trials justify the exclusion of a sham condition?

The answer to this question going forward again arguably lies in the UP-LIFT trial. All participants underwent intensive rehabilitation prior to transcutaneous spinal cord stimulation and, by all accounts, achieved benefits. This resolves, at least in part, the calculation of whether to include a sham control condition in future trials: all participants will benefit (i.e. rehabilitation alone), and some potentially more than others (i.e. active stimulation). The concern is not that one group will only be harmed, whatever that harm might be, including the notion of ‘time wasting’. Indeed, one would anticipate considerable enthusiasm among individuals with spinal cord injury for an opportunity to engage in rehabilitation with highly qualified therapists involved in clinical trials, even at the risk of not undergoing active stimulation.

Whatever the design is moving forward, acknowledging limitations is critically important in future trials. In the words of Dr John Ioannidis, this is important in order to place ‘research findings in context, interpreting the validity of the scientific work, and ascribing a credibility level to the conclusions of published research’.^9^ In transcutaneous spinal cord stimulation trials that do not include a sham control condition, limitations should acknowledge, at the very least, that some degree of improvement in function in individuals with spinal cord injury is related to factors underlying placebo effects.

## Conclusion

A 2009 position statement from the International Campaign for Cures of Spinal Cord Injury Paralysis,^10^ a consortium of not-for-profit organizations led by prominent researchers and clinicians involved in clinical trials whose goal was to expedite the discovery of cures for spinal cord injury, concluded that:

there is a significant placebo effect, especially in neurological diseases which functions such as sensation, spasms or residual movements can vary daily. The only accurate way to determine that a treatment is beneficial is to carry out a properly designed study with a placebo-treated ‘control’ group. Individuals or institutions selling therapy to-date have not carried out controlled trials with valid methods and outcome measures recorded by blinded observers.

This was written in the context of cell-based therapies and, in our opinion, carries forward to the case of neuromodulation.

There is ample reason to be excited about the potential of transcutaneous spinal cord stimulation and other neuromodulation techniques to restore neurological function in individuals with spinal cord injury. The results from the UP-LIFT trial have added to this excitement. However, in the absence of a trial demonstrating efficacy compared to a sham condition, the impact of placebo remains an important concern. Future studies are needed to design sham conditions that can be feasibly integrated into blinded clinical trials.
